# Editorial: Digital biomarkers in testing the safety and efficacy of new drugs in mental health: A collaborative effort of patients, clinicians, researchers, and regulators

**DOI:** 10.3389/fpsyt.2022.1107037

**Published:** 2023-01-11

**Authors:** Johanna Maria Catharina Blom, Cristina Benatti, Deborah Mascalzoni, Fabio Tascedda, Luca Pani

**Affiliations:** ^1^Department of Biomedical, Metabolic and Neural Sciences, University of Modena and Reggio Emilia, Modena, Italy; ^2^Department of Public Health and Caring Sciences, Centre for Research Ethics and Bioethics, Uppsala University, Uppsala, Sweden; ^3^Institute for Biomedicine, EURAC Research, Bolzano, Italy; ^4^Department of Life Science, University of Modena and Reggio Emilia, Modena, Italy; ^5^Department of Psychiatry and Behavioral Sciences, University of Miami School of Medicine, Miami, FL, United States; ^6^Relmada Therapeutics, Coral Gables, FL, United States

**Keywords:** digital biomarkers, digital phenotyping, digital endpoints, mobile sensing, remote assessment, cognition, psychiatry, data protection and privacy

The papers on this Research Topic demonstrate the necessity of including multiple viewpoints and approaches when considering the importance of digital biomarkers in testing the safety and efficacy of new drugs in mental health. In four excellent contributions, the concept of digital biomarkers in mental health is discussed from various angles. Papers range from defining the role of “bio” in digital biomarkers to explaining the idea beyond the “natural” part to their use in remote self-assessment and the need for regulatory rigor.

The challenge is 2-fold: most mental disorders, such as mood disorders, schizophrenia, and neurodegenerative disorders, such as Alzheimer's, are still classified and diagnosed by their observed symptoms, as little is known about their biological causes, and there is a lack of objective, measurable biological markers. This insufficient understanding poses significant challenges to developing new drugs to treat these diseases and how to test them. Second, when considering new approaches, such as bio-informed digital markers, they must live up to the quality standards of safety and efficacy established for the more traditional assessment strategies.

The ultimate goal is to design truly informative digital biomarkers and demonstrate that they fulfill this challenge. The use of digital biomarkers and digital monitoring has increased considerably and offers a new way to improve diagnostic and therapeutic research and development (1, 2). However, using digital biomarkers and digital phenotyping to inform novel targets for diagnostic and therapeutic intervention requires us to transcend their potential risks because of the lack of clear regulatory support.

The plan to attack this challenge requires serious collaboration between experts from different fields and calls for creating a platform that supports research across various areas that consider multiple angles. Collaborating with experts will help gain high-quality input into the design, testing, and validation of digital biomarkers and facilitate the development of new medicines for mental health.

We must agree on a common language to reach a consensus and the highest impact. When defining a biomarker, two aspects should be considered: its Identity: the name of the biomarker includes the specific analyte (e.g., fibrinogen), anatomic feature (e.g., joint angle), or physiological characteristic (e.g., blood pressure); and its Biologic Plausibility: the biological, physiological, or pathological pathway defining the association of the biomarker with the disease or condition of interest. Next, measurement methods should be clearly defined, and more importantly, we should consider using the (digital) biomarker.

Let's consider its use as a diagnostic marker. It is intended to detect or confirm the presence of a disease or a condition of interest or to identify individuals with a subtype or stage of the disease: For a perfect diagnostic biomarker test, all patients with the disease or disease subset would be detected (100% sensitivity), and no patients without the disease would be diagnosed (100% specificity). In practice, no biomarker test has a perfect clinical and analytical performance, which is even more problematic for digital biomarkers.

Biomarkers can also be used as pharmacodynamic, predictive, prognostic, and surrogate markers, including reasonably likely surrogate endpoints. In all cases, acceptable tradeoffs among performance characteristics, such as sensitivity, and specificity, must be considered and will depend, for example, on the potential harms of false positive and false negative results.

Suppose their uses are intended as monitoring biomarkers. In that case, we want to use them repeatedly for assessing the status of a disease or medical condition or for evidence of exposure to (or effect of) a medical product, disease progression, including the occurrence of new disease effects, worsening of previously existing abnormalities, or change in disease severity or specific exceptions, the response of a disease or condition to treatment, either favorable or unfavorable. Biomarkers with this scope include safety biomarkers, pharmacodynamic/response biomarkers, or prognostic biomarkers. Digital biomarkers will have to guarantee the same performance qualities.

About these important defining issues, the review article by Montag et al. outlines the existing difficulties regarding the definition of digital biomarkers and asks how much biology needs to be in a Digital Biomarker to be reliable. Succinctly, the paper exposes, on the one hand, the current lack of how to define a biomarker; on the other hand, it provides an overview of various definitions and characterizes direct (narrow) or indirect (broad) concepts of digital biomarkers. They explain that, from their perspective, digital biomarkers represent a more direct (or little) concept describing digital footprints as being directly linked to biological variables, such as molecular, genetic, epigenetic ones, and brain imaging. In contrast, digital prints are linked to human behavior that may act as latent variables indirectly related to biological variables, representing more indirect notions of what a digital biomarker constitutes.

Building on this last concept, the paper by Seixas et al. affronts traditional biomarkers' lack of predictive ability to determine cognitive functioning and changes in older adults leading to misdiagnosis and inappropriate treatment plans. They explored whether a digital neuro signature (DNS-br) biomarker efficiently predicted global cognitive functioning and change over time in cognitively impaired older adults and compared the effect size of the DNS-br biomarker on global cognitive functioning to traditional imaging and genomic biomarkers. Their study shows that a digital biomarker predicted cognitive functioning and change where established biomarkers failed to do so reliably. The Altoids tool proved highly useful in screening, prediction, prevention, and symptom monitoring, identifying older adults at risk for mild cognitive impairment and dementia.

The paper by Atkins et al. goes a step further. It contributes to the growing literature on wearable and “nearly” technology sensors, surveys, games, and computer mouse movements as digital biomarkers to infer cognitive status (Atkins et al.). Rather than traditional in-house evaluation, their study describes the feasibility, reliability, and sensitivity of remote self-administration of brief cognitive tests in older adults with and without subjective cognitive decline using an FDA/EMA-compliant testing platform. Both the paper by Seixas et al. and Atkins et al. show how to move the field of digital biomarkers in brain health forward. Combining digital health technologies with home-based digital technologies will help to improve the early detection of cognitive decline, identify which cognitive domains are affected in real-life settings, and study the precise impact of treatments on specific cognitive functions. However, to fully embrace digital biomarkers, we need to create a context where their use is regulated most satisfactorily.

Among these digital biomarkers, in line with recent regulatory guidelines (*US Food Drug Administration. Digital Health Technologies for Remote Data Acquisition in Clinical Investigations Guidance for Industry, Investigators, and Other Stakeholders. Food and Drug Administration (US)* (2022). Available online at: https://www.fda.gov/regulatory-information/search-fda-guidance-documents) Gallucci et al. presented an innovative analysis of those collected by Nurosene's NURO app (nurosene.com) a smartphone application that gathers and analyzes active self-report metrics from users, empowering them with data-driven health machine intelligence. Exploratory results using a variational autoencoder (VAE) suggested that much of the variability of the 12-dimensional data could be accounted for by two approximately uncorrelated latent variables: one pertaining to stress and sleep, and the other pertaining to exercise and diet. Subsequent modeling of the data using exploratory and confirmatory factor analyses (EFAs and CFAs) found that optimal data fits consisted of four factors, namely exercise, diet, stress, and sleep. Covariance values were high between exercise and diet, and between stress and sleep, but much lower between other pairings of non-identical factors. Overall, these results significantly reduce the apparent complexity of the response data, suggesting the possibility of applying predictive analytics in future efforts (Seixas et al.).

To this end, Parziale and Mascalzoni (3) explored issues related to data protection. They showed the possible risk of user-generated data as “digital biomarkers” when sharing mental health data exposes patients to discrimination, resulting in reduced participation and trust. An essential way of avoiding this trend is to implement an appropriate data governance system with a clear and transparent allocation of data protection duties and responsibilities among the actors involved in the process. This should include appropriate measures to avoid stigmatization and the increase of disparities (https://pubmed.ncbi.nlm.nih.gov/35688129/) both for the collection and potential use of data and at the same time promotes equity for precision health.

Their paper examines the lack of precise data protection regulation and proposes practical recommendations for a comprehensive approach that allows the integration of digital biomarkers in psychiatric research in an ethical, legal, and trusted ecosystem.

Finally, incorporating digital biomarkers in our clinical and research approach facilitates clinical diagnosis and trial design. It helps accelerate the development of treatments for neuropsychiatry conditions such as schizophrenia, Alzheimer's, and major depression. It will also pave the way for a cultural change, stimulate discussion regarding approaches to data quality in medical research and drug development and assist in defining quality management systems that help reach consensus among stakeholders regarding quality recommendations for research and clinical use. Current research suggests that there is an urgent need to incorporate digital biomarkers into patient care and monitoring, especially in the realm of mental disease, because they are unrestricted by time and place, offer immediate access to data and intermediate endpoints, reduce stressful visits, remove barriers to access to care (fear, isolation), stimulate patient empowerment, and, most importantly, help in the identification of high-risk patients and their risk stratification.

The digital era, which includes digital biomarkers, phenotyping, and surveillance, falls entirely in the realm of precision medicine and underlines the importance of incorporating a digital biologically informed approach to enhance our biological understanding of mental health and patient care in general. Indeed, we must work on gaining regulatory approval for digital biomarkers in mental health as we have to optimize clinical trials incorporating an ethically informed digital approach and work to overcome the present shortcoming. But it is undeniable that we are on the right track.

## Author contributions

All authors contributed to the content of the editorial, JB final editing.
